# Adsorption and Photocatalytic Processes of Mesoporous SiO_2_-Coated Monoclinic BiVO_4_

**DOI:** 10.3389/fchem.2018.00415

**Published:** 2018-09-19

**Authors:** Duangdao Channei, Auppatham Nakaruk, Wilawan Khanitchaidecha, Panatda Jannoey, Sukon Phanichphant

**Affiliations:** ^1^Department of Chemistry, Faculty of Science, Naresuan University, Phitsanulok, Thailand; ^2^Research Center for Academic Excellence in Petroleum, Petrochemicals and Advanced Materials, Naresuan University, Phitsanulok, Thailand; ^3^Department of Industrial Engineering, Faculty of Engineering, Naresuan University, Phitsanulok, Thailand; ^4^Centre of Excellence for Innovation and Technology for Water Treatment, Naresuan University, Phitsanulok, Thailand; ^5^Department of Civil Engineering, Faculty of Engineering, Naresuan University, Phitsanulok, Thailand; ^6^Department of Biochemistry, Faculty of Medical Science, Naresuan University, Phitsanulok, Thailand; ^7^Materials Science Research Center, Faculty of Science, Chiang Mai University, Chiang Mai, Thailand

**Keywords:** composite materials, photocatalysis, BiVO_4_, BET isotherms, SiO_2_

## Abstract

The silicon dioxide (SiO_2_)–coated bismuth vanadate (BiVO_4_) composites as visible–driven–photocatalysts were successfully synthesized by the co–precipitation method. The effects of SiO_2_ coating on the structure, optical property, morphology and surface properties of the composites were investigated by X–ray diffraction (XRD), UV–visible diffuse reflectance spectroscopy (DRS), transmission electron microscopy (TEM) and Brunauer–Emmette–Teller (BET) measurements. The photocatalytic activity of monoclinic BiVO_4_ and BiVO_4_/SiO_2_ composites were evaluated according to the degradation of methylene blue (MB) dye aqueous solution under visible light irradiation. The SiO_2−_coated BiVO_4_ composites showed the enhancing photocatalytic activity approximately threefold in comparison with the single phase BiVO_4_.

## Introduction

Nowadays, the advanced oxidation process is known as an effective method for water purification and wastewater treatment. One of the most famous advanced oxidation process is heterogeneous photocatalysis; the contaminant (i.e., organic compounds) containing in the water and wastewater is finally degraded to carbon dioxide (Legrini et al., 1993; Mukherjee and Ray, 1999). This process can remove the organic contaminant perfectly and does not generate the second contaminant (i.e., sludge and other organic compounds) which are required the further treatment and disposal. According to the heterogeneous photocatalysis, the titanium dioxide (TiO_2_) has been played a role as the important catalyst to promote the photocatalytic activity. Due to its wide band gap of 3.2 eV, the photocatalyst of TiO_2_ is typically activated under the UV light (the wavelength <390 nm is required), which accounts for 45–50% of solar radiation (Linsebigler et al., 1995; Bahnemann et al., 2007; Devipriya et al., 2012). This theoretical fact becomes the limitation and non-cost-effectiveness of actual photocatalytic system for purifying the water at the site.

Another catalyst of monoclinic bismuth vanadate (BiVO_4_) has been proposed to overcome the drawback of photocatalytic system using TiO_2_ and together with enhance the photocatalytic activity during implementation. Since BiVO_4_ has narrow band gaps of 2.4 to 2.8 eV (Kudo et al., 2001; Xie et al., 2006; Li et al., 2008), this photocatalyst can be activated by the visible light and consequences the effective use of solar energy. However, the low specific surface area and poor surface textural property are the significant disadvantages of using BiVO_4_ as the catalyst. Its low surface area and adsorption capacity cause the low efficiency of photocatalytic system for organic contaminant removal and also the long treatment period required. Therefore, the increase in specific surface area of BiVO_4_ catalyst is necessary prior to imply the photocatalytic system to the actual wastewater.

Recently, alternative composite materials have been synthesized by combining metal oxide and porous materials (i.e., alumina, silica, zeolites, carbon black, charcoal) (Belessi et al., 2007; Wang et al., 2012; Xing et al., 2016) with the aim of improving the specific surface area, pore structure, and photocatalytic activity of catalysts (Gan et al., 2003; Kimura et al., 2003). For example, the enhancement of Ag–doped TiO_2_ photocatalytic activity was suggested by adding the mesoporous SiO_2_; the excellent efficiency of methyl orange (MO) removal was achieved by 2.5 h (Roldan et al., 2015). The increasing adsorption capacity of TiO_2_ catalyst was observed when the catalyst was combined with SiO_2_; the adsorption capacity was increased (Hu et al., 2012). The SiO_2_ addition also enhance the separation rate of electron–hole pairs under UV excitation. Further, the deposition of gold nanoparticles (Au) on the porous SiO_2_-WO_3_ composite can enhance the methylene blue (MB) adsorption capacity; the adsorption capacity of Au–SiO_2_-WO_3_ was greater than SiO_2_-WO_3_ and WO_3_ respectively (DePuccio et al., 2015). The complete MB removal was achieved by 300 min under visible light, and the fast kinetic of MB removal was found in Au–SiO_2_-WO_3_ catalyst, following by Au–WO_3_ and WO_3_ catalysts.

As all the above mentions, this study aimed to improve the surface morphology and photocatalytic activity of BiVO_4_ catalyst by coating SiO_2_. Various analytical techniques including X–ray diffraction (XRD), transmission electron microscopy (TEM), Brunauer–Emmett–Teller (BET) and UV–vis diffuse reflectance spectra (DRS) were used to clarify the better property of BiVO_4_/SiO_2_ composite rather than BiVO_4_ and SiO_2_. Further, the performance of BiVO_4_/SiO_2_ composites on wastewater treatment was preliminary studied in the batch test under visible light irradiation, and its performance was compared to the other two materials of BiVO_4_ and SiO_2_.

## Experimental procedure

All chemicals used were of analytical grade and were used as received without any further purification. The chemicals including tetraethyl orthosilicate (TEOS), bismuth (III) nitrate pentahydrate [Bi(NO_3_)_3_·5H_2_O], ammonium metavanadate (NH_4_VO_3_), methylene blue powder, sodium hydroxide pellet (NaOH), ammonia solution (28%) and nitric acid (37% HNO_3_) were obtained from Sigma-Aldrich. All solutions were prepared with deionized water.

### Preparation of SiO_2_ particles

SiO_2_ particles were prepared by the sol–gel method. Ammonia solution (28%) was added in 100 mL of a mixed solution of absolute ethanol/DI water (80: 20 v/v) and stirred under ultrasonic dispersion for 60 min. Then, 20 mL of tetraethyl orthosilicate (TEOS) was added drop by drop to the mixed solution and stirred for 120 min at room temperature. After the reaction was homogenized, the fine particles were separated by centrifugation with typical rotating speed of 6,000 rpm for 15 min, washed by DI water and dried at 80°C for 24 h in a hot air oven. Fine particles of SiO_2_ were obtained as a white powder following heat treatment at 500°C for 1 h in ambient.

### Preparation of monoclinic BiVO_4_ and SiO_2_-coated BiVO_4_ composites

Monoclinic BiVO_4_ were obtained by the co–precipitation method. Firstly, 12 mmol of bismuth (III) nitrate pentahydrate [Bi(NO_3_)_3_·5H_2_O] and the same volume of ammonium metavanadate (NH_4_VO_3_) were dissolved in 100 mL of 2 M nitric acid (HNO_3_) under vigorous stirring. The pH of the mixed solution was adjusted to 9 by adding 3 M sodium hydroxide (NaOH). The yellow precipitate was then separated by centrifugation at 6,000 rpm for 15 min, washed thoroughly with distilled water and ethanol and finally dried in a hot air oven at 80°C for 24 h. Crystalline monoclinic BiVO_4_ was formed after calcination at 550°C for 4 h.

BiVO_4_-coated SiO_2_ composites were also prepared by the same method for comparison with an additional step of adding SiO_2_ powder to 100 mL of 2 M HNO_3_.

### Photocatalytic reaction

Photocatalytic activities of the BiVO_4_, SiO_2_ and BiVO_4_/SiO_2_ composites were evaluated through degradation of methylene blue (MB) dye as a model organic pollutant under visible light. A total of 0.20 g of photocatalyst was added to 100 mL MB aqueous solution (initial concentration C_0_ = 20 ppm) under magnetic stirring in darkness for 60 min to achieve adsorption–desorption equilibrium. The system was irradiated by three 18 W halogen lamps (Essential MR, Philips, Thailand) to investigate photocatalytic degradation. Reduction of MB concentration over time (C_t_) was recorded every 15 min by measuring the intensity change of the characteristic absorption peak at 664 nm using UV–vis double beam spectroscopy (UV−6100, Mapada).

### Characterisation

Crystal phase and structure of the prepared samples were characterized by powder X–ray diffraction (XRD, Philips X'Pert MPD) using Cu K_α_ (λ = 1.54056 Å) radiation. Morphological changes in the composite materials were monitored by transmission electron microscopy (TEM, JSM−2010, JEOL). Brunauer–Emmett–Teller (BET) measurements (Adtosorb 1 MP, Quantachrome) were performed to compare the specific surface area of the BiVO_4_ and BiVO_4_/SiO_2_ composites. Measurement of UV–vis diffuse reflectance spectroscopy (DRS UV–vis, Shimadzu UV−3101PC) was carried out at room temperature to detect reflectance and absorbance spectra.

## Results and discussion

**Figure 1:**
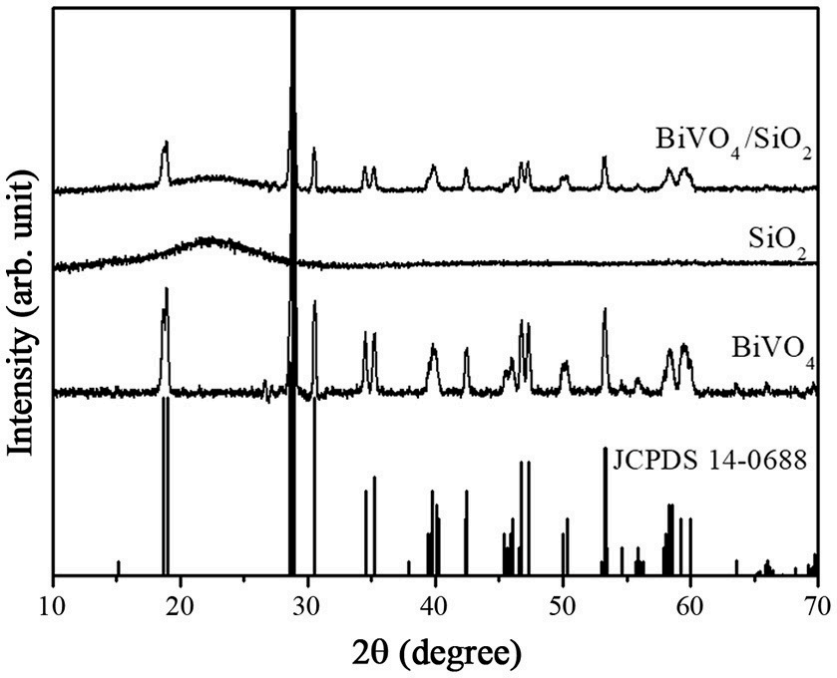
XRD patterns of as–prepared BiVO_4_, SiO_2_, and BiVO_4_/SiO_2_.

In Figure 1, the broad XRD peak at 2θ = 22–23° corresponded to the amorphous SiO_2_. The XRD pattern of BiVO_4_ without SiO_2_ was assigned to the standard monoclinic BiVO_4_ (JCPDS no. 14–0688) (Gotić et al., 2005). After coating BiVO_4_ with SiO_2_, the diffraction peaks matched well with the pure phase monoclinic BiVO_4_ and no peaks of any other phases or impurities were recorded. However, the diffraction intensity of BiVO_4_ decreased after coating SiO_2_, because the amorphous substance had the negative effect on crystallinity. Alternatively, self–doped Si^4+^ ions in the BiVO_4_ crystal structure might cause the decreasing crystallinity of BiVO_4_/SiO_2_ composites, and resulted in the broader peaks of the composite samples, which are similar to those reported by Phanichphant et al. (2016) for the binary composite CeO_2_/SiO_2_ photocatalyts and Kumar et al. for TiO_2_/SiO_2_ nanocomposites in solar cell applications (Arun Kumar et al., 2012).

**Figure 2:**
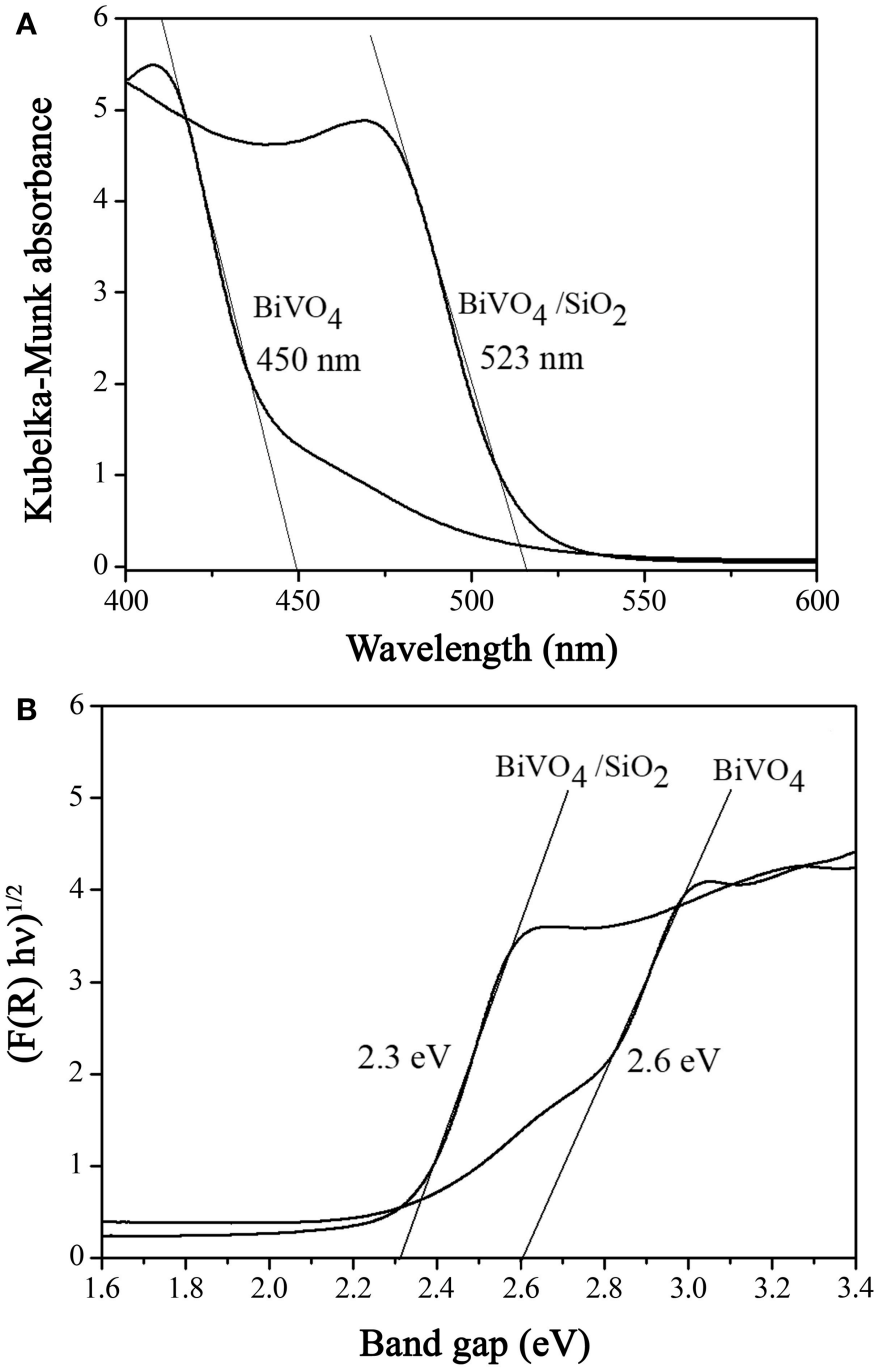
**(A)** Diffuse reflectance UV–visible spectra and **(B)** the plot of adsorption function vs. photon energy for determination of band gap (E_g_).

As shown in Figure 2a, the BiVO_4_ demonstrated the absorption edge of the visible region at 450 nm, corresponding to the optical band gap (E_g_) of 2.60 eV which was calculated by the Kubelka–Munk function (see Figure 2b) (Sirita et al., 2007). Compared to BiVO_4_/SiO_2_ composites, the value of the graph intercept was estimated at 2.30 eV, corresponding to the strong absorption edge in the visible region at 523 nm. The band gap energy of BiVO_4_ decreased from 2.60 to 2.30 eV in the composite materials, due to the influence of Si^4+^ ions doping into the lattice of BiVO_4_ which created the abundant doping energy levels. The estimated band gap values in this study was similar to those of BiVO_4_ reported by Jiang et al. (2012), who prepared the BiVO_4_ photocatalysts with different morphologies using the hydrothermal method. Liu et al. (2015) observed that the band gap energy of BiVO_4_/SiO_2_ catalyst estimated to be 2.32 eV, which was almost the same as that of calculate by this study.

**Figure 3:**
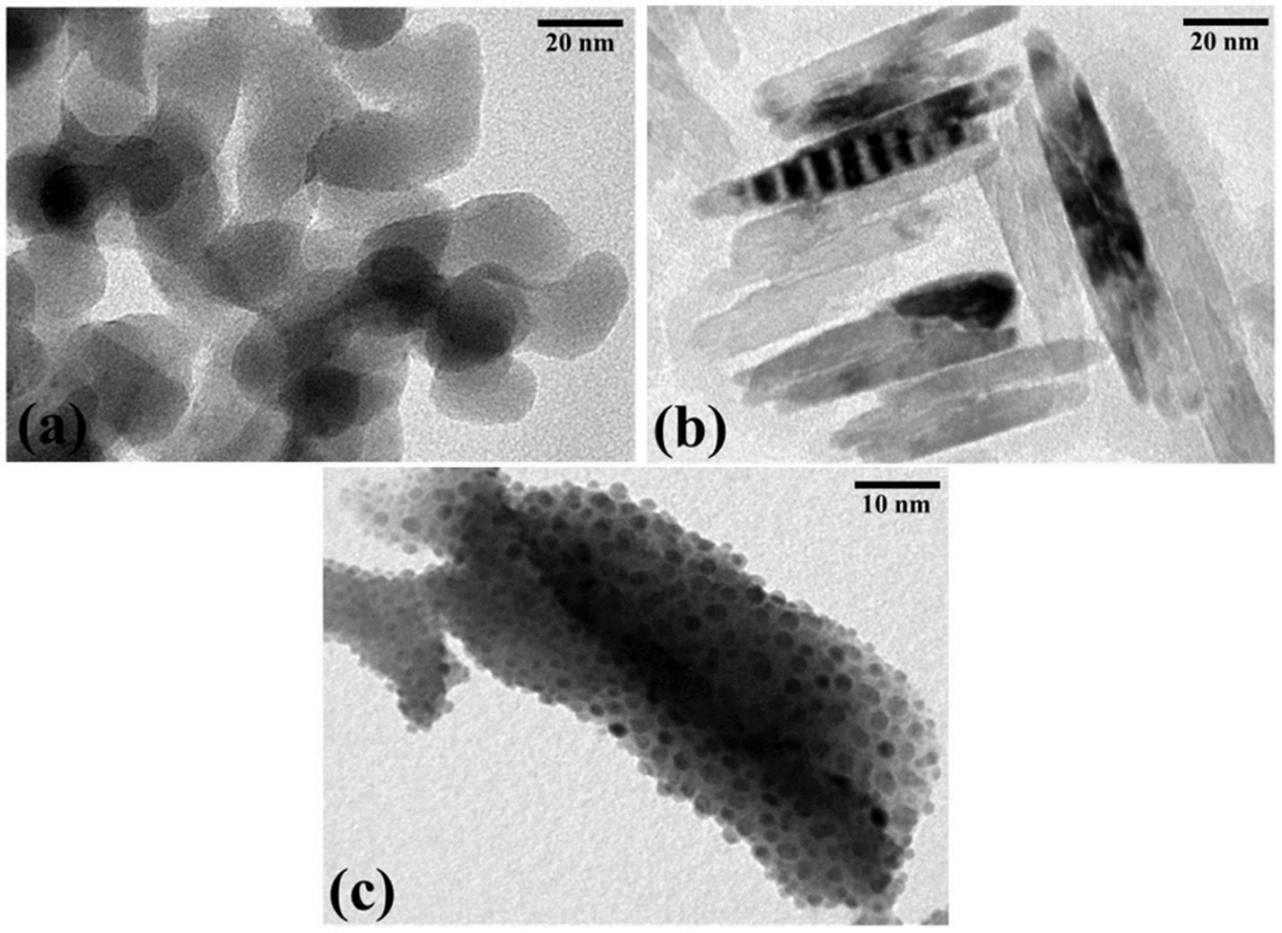
TEM images of **(a)** SiO_2_, **(b)** BiVO_4_, and **(c)** SiO_2_-coated BiVO_4_.

The TEM images of SiO_2_, BiVO_4_ and BiVO_4_/SiO_2_ composites are presented in Figure 3. The SiO_2_ image shows the aggregation of spherical–shaped particles with diameters ranging of 20–30 nm (Figure 3a), while Figure 3b shows the rod–like nanostructures of monoclinic BiVO_4_ with the diameter of 10 nm and the length of 60 nm. Typical TEM images are used for characterizing the composite materials and proving the heterojunction formation between BiVO_4_ and SiO_2_, which demonstrated that the rod–like BiVO_4_ core was covered by the SiO_2_ particles growing on the surface (Figure 3c).

**Figure 4:**
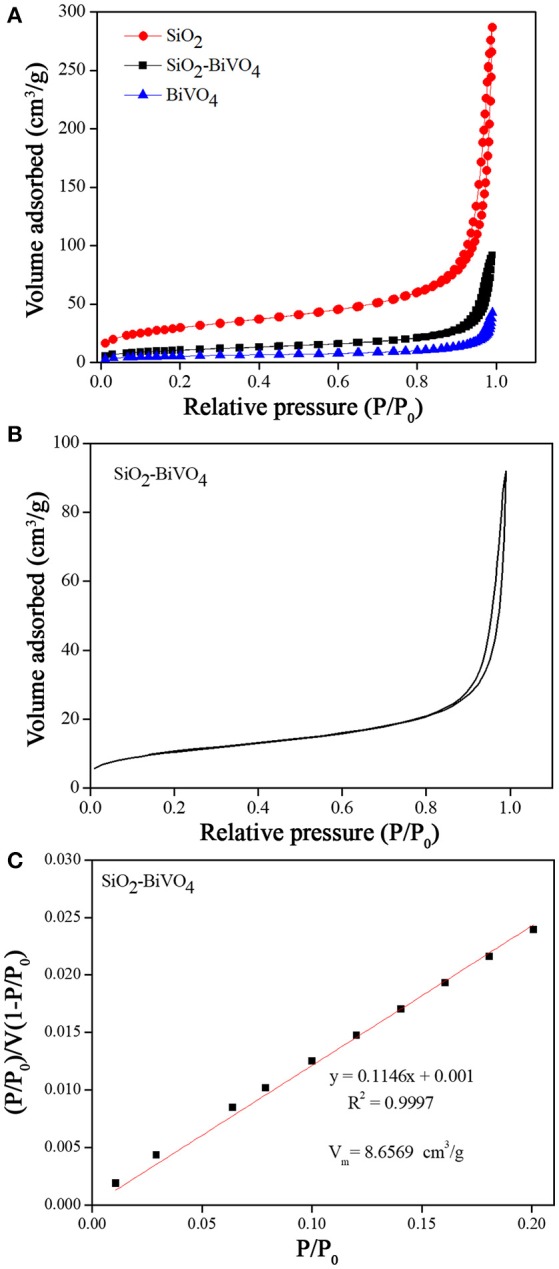
**(A)**, **(B)** N_2_ adsorption-desorption isotherms, and **(C)** BET linear plot of relative pressure.

The N_2_ adsorption-desorption isotherms (Figure 4a) show that the N_2_ adsorption of BiVO_4_/SiO_2_ composites were relatively higher than that of the pure BiVO_4_, however the value was much lower than that of the SiO_2_. The specific surface areas of SiO_2_, BiVO_4_/SiO_2_ composites, and BiVO_4_ were found to be 106.9959, 37.6851, and 19.4964 m^2^/g, respectively. In the meanwhile, the pore size was calculated by using the BJH method, and the results were 9.0316, 11.0776, and 11.8111 nm for SiO_2_, BiVO_4_/SiO_2_, and BiVO_4_ respectively (as summarized in Table 1). The surface area and pore size are positively related to the photocatalytic activity, therefore the photocatalytic activity of BiVO_4_/SiO_2_ composites were higher than that of pure BiVO_4_. Even though the surface area of SiO_2_ was higher than the BiVO_4_/SiO_2_ composite, the adsorption of pollutant by SiO_2_ with high specific surface area have only the ability to transfer pollutants to alternative phases, but not completely get rid of them. Therefore, the photocatalytic process based on using the hydroxyl radicals is required in this study.

**Table 1:** Surface properties of the prepared samples.

**Sample**	**Specific surface area (m^2^/g)**	**Average pore size diameter (nm)**
SiO_2_	106.9959	9.0316
BiVO_4_/SiO_2_	37.6851	11.0776
BiVO_4_	19.4964	11.8111

Figure 4b shows the N_2_ adsorption-desorption isotherms of BiVO_4_/SiO_2_ composites in the relative pressure (P/P_0_) range 0.00–1.00. The curve exhibited Type IV isotherm characteristic with a small hysteresis loop at the relative pressure of 0.80–1.00. This indicated the existence of mesopores in the sample with the pore diameter ranging of 2–50 nm (Brunauer et al., 1940; Bae et al., 2010).

The information from the isotherm can be used to determine the specific surface area from the mathematical relations in Equation (1) and Equation (2) below (Itodo et al., 2010; Thommes et al., 2015)

(1) $$\begin{array}{r} \frac{\text{P}/{\text{P}}_{0}}{\text{V}\left(1-\text{P}/{\text{P}}_{0}\right)}=\frac{1}{{\text{V}}_{m}C}+\frac{\left(\text{C}-1\right)}{{\text{V}}_{m}C}\frac{\text{P}}{{\text{P}}_{0}} \end{array}$$

where,

P_0_, Initial pressure of N_2_; P, Equilibrium pressure of N_2_ adsorption; V_m_, Monolayer capacity; V, Amount of N_2_ adsorbed at standard temperature and pressure (STP).

(2) $$\begin{array}{r} \text{Specific Surface area}=\frac{{\text{V}}_{\text{m}}N_{a}\times A}{\text{m}\times 22400} \end{array}$$

where,

A, Cross-sectional area of the adsorbed N_2_; m, Adsorbate molecular weight; N_a_, Avogadro's number.

The intercept and slope of the plot in Figure 4c were used to calculate the maximum volume of gas adsorbed at the monolayer (V_m_), it was 8.6569 cm^3^/g. The specific surface area was also calculated via the V_m_ value (see Equation 2). The result showed that the surface area of BiVO_4_/SiO_2_ composites was 37.6851 m^2^/g.

**Figure 5:**
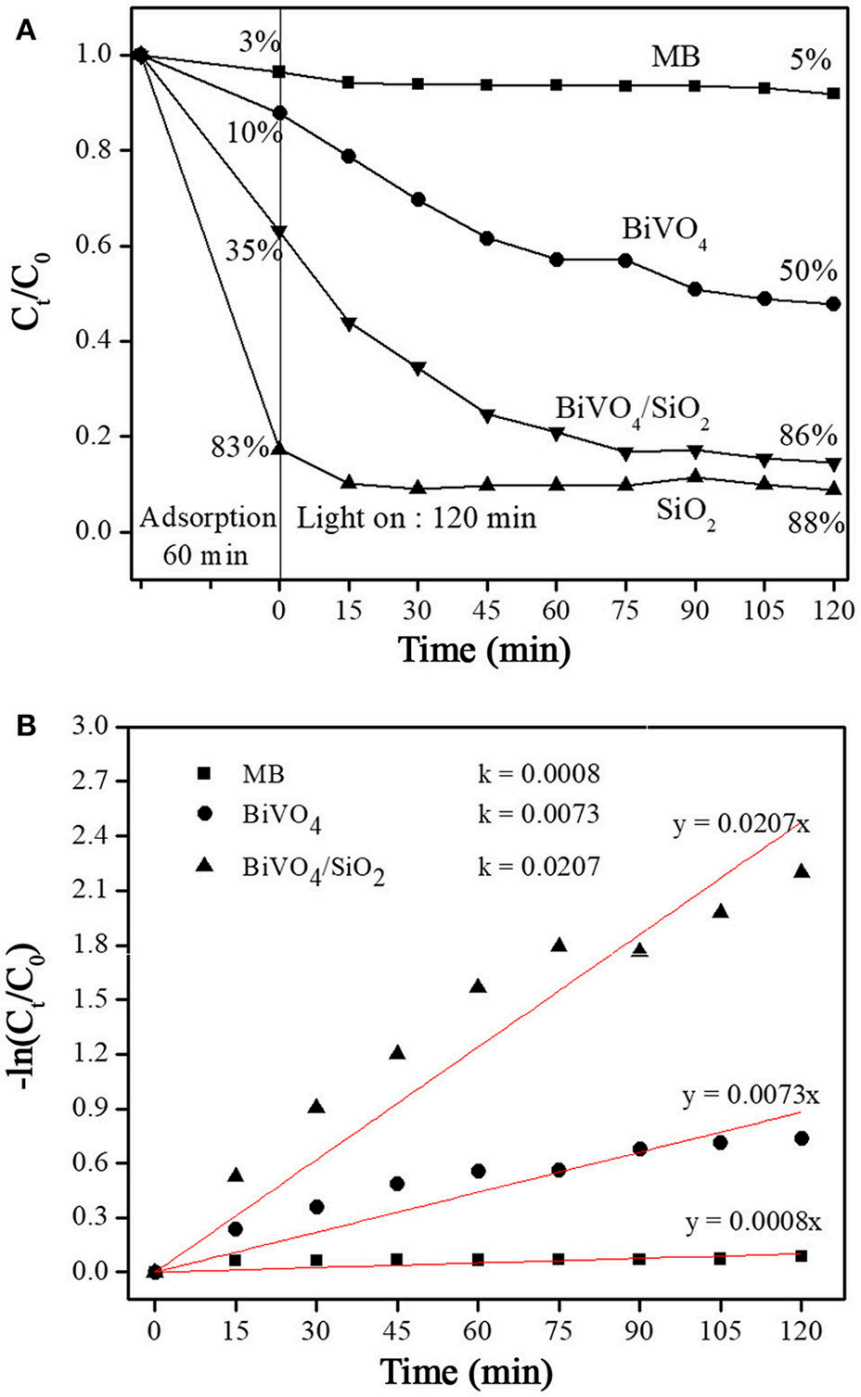
MB concentration changes with irradiation time **(A)** C_t_/C_0_ and **(B)** –ln C_t_/C_0_.

Figure 5a presents the degradation efficiency of MB as a function of C_t_/C_0_ and visible irradiation time. The C_0_ was the initial concentration of MB before irradiation and C_t_ was the MB concentration at the interval irradiation time (t, min). For using the SiO_2_ as catalyst, the MB was removed of 83% under the dark adsorption, and only 5% of MB was further degraded under the visible light. For using the single phase monoclinic BiVO_4_, the MB was removed around 10% under the dark adsorption, and 40% of MB was further degreased under the visible light irradiation. When the BiVO_4_/SiO_2_ composites was used, the MB removal efficiency reached 35 and 86% under the dark adsorption and visible light irradiation. As above explanation, the specific surface area of BiVO_4_/SiO_2_ composites were increased from BiVO_4_, due to the SiO_2_ coating. The increasing specific surface area resulted in the high adsorption of MB molecules during 60 min of the darkness, and then the adsorbed MB was continuously degraded by photocatalytic activity during visible light. These results illustrated that the photocatalytic activity of BiVO_4_ was enhanced by coating the SiO_2_ particles.

The kinetics of MB degradation was analyzed using the pseudo–first order model, which was given in Equation (3) (Yetim and Tekin, 2017). In Figure 5b, the correlation of–ln C_t_/C_0_ and t were positive with linear equation; the kinetic constant (k) were 0.0073 min^−1^ for BiVO_4_ and 0.0207 min^−1^ for BiVO_4_/SiO_2_ composites. The kinetic constant of MB degradation using BiVO_4_/SiO_2_ composites was approximately threefold higher than that using the single phase BiVO_4_.

(3) $$\begin{array}{r} -\text{ln}\left({\text{C}}_{\text{t}}/C_{0}\right)=kt \end{array}$$

where k is the apparent rate constant of the pseudo–first order reaction (min^−1^).

**Figure 6:**
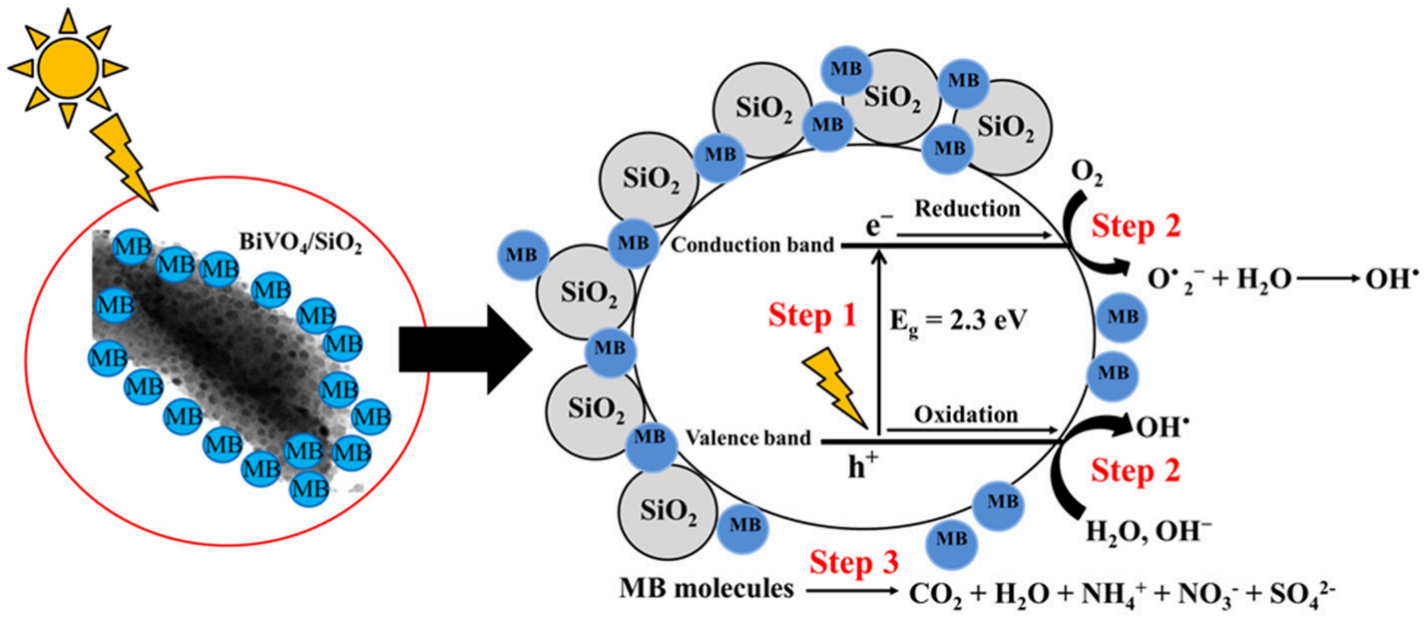
The proposed mechanism of photogenerated charge carriers of BiVO_4_ in BiVO_4_/SiO_2_ heterojunction.

Since the photocatalytic degradation of dyes is associated with dye adsorption onto the surface of BiVO_4_/SiO_2_. Furthermore, photocatalytic degradation occurs at or near the surface of the catalyst rather than in the bulk solution. Thus the higher photocatalytic activity of BiVO_4_/SiO_2_ is consistent with the higher adsorption of MB on the surface of BiVO_4_/SiO_2_ photocatalyst. Mesoporous SiO_2_ adsorbent enriches the MB molecules around the BiVO_4_ surface as shown in Figure 6 and the visible–light photocatalytic activity of the BiVO_4_ interface in the composite materials is then excited to generate electrons (e–) and holes (h^+^). Subsequently, photoexcited electrons in the valance band and hole in the conduction band of BiVO_4_ react with oxygen, water and hydroxide ions to produce free superoxide radicals (O${}_{2}^{-•}$) and hydroxyl radicals (OH^•^) as the main active oxidizing species, which then react with MB molecules during the photocatalytic process (Lin et al., 2014; Zhou et al., 2014). The final products of MB aqueous solution photocatalytic degradation are oxidized to CO_2_, H_2_O, CO_2_, NH${}_{4}^{+}$, NO${}_{3}^{-}$, and SO${}_{4}^{2-}$ (Houas et al., 2001; Luan and Hu, 2012).

## Conclusions

BiVO_4_/SiO_2_ composites consisting of spherical SiO_2_ particles coated on BiVO_4_ nanorods were successfully prepared by co–precipitation. The composites exhibited higher photocatalytic activity compared to single monoclinic BiVO_4_ by degrading MB under visible–light irradiation due to the greater surface area of mesoporous SiO_2_. Fabrication of heterogeneous semiconductors using mesoporous materials can produce promising alternative photocatalysts for wastewater treatment under light irradiation by combining adsorption and photocatalytic processes.

## Author contributions

DC designed and performed the experiments and wrote the manuscript. SP, AN advised the data analysis and edited manuscript. PJ and WK advised the data analysis. All authors reviewed the approved the manuscript.

### Conflict of interest statement

The authors declare that the research was conducted in the absence of any commercial or financial relationships that could be construed as a potential conflict of interest.
